# Daily Sitting for Long Periods Increases the Odds for Subclinical Atheroma Plaques

**DOI:** 10.3390/jcm10061229

**Published:** 2021-03-16

**Authors:** Jose Luis Perez-Lasierra, Martin Laclaustra, Pilar Guallar-Castillón, Jose Antonio Casasnovas, Jose Antonio Casajús, Estibaliz Jarauta, Alejandro Gonzalez-Agüero, Belen Moreno-Franco

**Affiliations:** 1 Department of Physiatry and Nursing, Universidad de Zaragoza, 50009 Zaragoza, Spain; jlperez@unizar.es (J.L.P.-L.); joseant@unizar.es (J.A.C.); alexgonz@unizar.es (A.G.-A.); 2 GENUD (Growth, Exercise, Nutrition and Development) Research Group, 50009 Zaragoza, Spain; 3 Department of Medicine, Psiquiatry and Dermatology, Universidad de Zaragoza, 50009 Zaragoza, Spain; martin.laclaustra@unizar.es (M.L.); jacasas@unizar.es (J.A.C.); estijarauta@gmail.com (E.J.); 4 Instituto de Investigación Sanitaria Aragón, Hospital Universitario Miguel Servet, 50009 Zaragoza, Spain; 5 CIBERCV Instituto de Salud Carlos III, 28029 Madrid, Spain; 6 Department of Preventive Medicine and Public Health, School of Medicine, Universidad Autónoma de Madrid-IdiPaz, 28029 Madrid, Spain; mpilar.guallar@uam.es; 7 CIBERESP Instituto de Salud Carlos III, 28029 Madrid, Spain; 8 IMDEA-Food Institute, CEI UAM + CSIC, 28049 Madrid, Spain; 9 Department of Microbiology, Pediatrics, Radiology and Public Health, Universidad de Zaragoza, 50009 Zaragoza, Spain

**Keywords:** sitting time, sedentary behavior, subclinical atherosclerosis, cardiovascular disease

## Abstract

Sedentarism is a risk factor for cardiovascular disease (CVD), but currently it is not clear how a sedentary behavior such as long sitting time can affect atherosclerosis development. This study examined the relationship between sitting time and the prevalence of carotid and femoral subclinical atherosclerosis. A cross-sectional analysis based on a subsample of 2082 participants belonging to the Aragon Workers’ Health Study was carried out. Ultrasonography was used to assess the presence of plaques in carotid and femoral territories; the validated Spanish version of the questionnaire on the frequency of engaging in physical activity used in the Nurses’ Health Study and the Health Professionals’ was used to assess physical activity and sitting time; and demographic, anthropometric, and clinical data were obtained by trained personnel during the annual medical examination. Participants were categorized into <9 h/day and ≥9 h/day sitting time groups. After adjusting for several confounders, compared with participants that remain seated <9 h/day, those participants who remain seated ≥9 h/day had, respectively, OR = 1.25 (95%CI: 1.01, 1.55, *p* < 0.05) and OR = 1.38 (95%CI: 1.09, 1.74, *p* < 0.05) for carotid and any-territory plaque presence. Remaining seated ≥9 h/day is associated with higher odds for carotid and any-territory plaque presence independently of physical activity levels and other cardiovascular risk factors.

## 1. Introduction

The first functional and pathological changes of atherosclerosis appear early in the youth and it slowly progresses all through life [1] until it manifests as clinical cardiovascular disease (CVD), which occurs mainly from the fifth decade of life on, creating a remarkable personal and social burden. Acting upon lifestyle and behavior is one of the keystones in CVD prevention and control [1]. Sitting time has been identified in the last decade as a lifestyle factor that increases the prevalence of CVD and its risk factors, independent of physical activity performed [2]. In fact, there is evidence that the sitting time and physical inactivity affects cardiovascular physiology and metabolism differently, each with a specific biological effect [3,4,5]. While physical inactivity has been ranked as the fourth global leading cause of death [6], sitting time has been described as an independent major mortality risk factor, responsible for 3.8% of all deaths [7]. Prospective studies evidenced that two hours per day of additional sitting time increases the risk of cardiovascular mortality and cardiovascular events on 5% and 17%, respectively, regardless of the physical activity level [2].

Adopting the sitting position has been associated with changes in carotid artery hemodynamics, and these, in turn, have been hypothesized to lead to atherosclerosis in that territory [8]. Despite evidence on long term effects of sitting time, being able to show early deleterious effects is important in order to reinforce the knowledge on the causal chain, and to provide solid evidence to health promotion campaigns, which should intervene early in life. However, although epidemiological research discovered the association between sitting time and several CVD risk factors such as incident type 2 diabetes [9], inflammatory markers [10,11], and poor lipid profile [12], data about the effect of sitting time on subclinical atherosclerosis are very scarce. Few studies have evaluated the association of sitting time or sedentary time with the presence of plaques in the carotid arteries and they could not demonstrate statistically significant results [13,14,15]. Thus, relying on a wide sample, meticulously studied for subclinical atherosclerosis, we aim to re-examine the association of sitting time with the presence of carotid and femoral atherosclerosis plaques on male workers from a factory in Spain who belong to the Aragon Workers’ Health Study (AWHS) cohort.

## 2. Materials and Methods

### 2.1. Study Design and Participants

This cross-sectional analysis was carried out in a subsample of participants belonging to the AWHS [16]. The AWHS is a prospective cohort aiming to investigate the determinants of the development and progression of metabolic abnormalities and subclinical atherosclerosis in 5678 workers of a car-manufacturing factory, free of clinical CVD, and recruited between 2009 and 2012. From 2011 to 2014, 2646 participants between 39 and 59 years of age accepted detailed explorations when they were additionally invited to undergo subclinical atherosclerosis measurements, and to complete diet, behavior, and lifestyle questionnaires. We excluded women (*n* = 132) and those with missing data on relevant variables (*n* = 432), so the final sample was composed of 2082 men. The study was approved by the Clinical Research Ethics Committee of Aragon (CEICA). All participants provided written informed consent.

### 2.2. Subclinical Atherosclerosis Imaging

A Philips IU22 ultrasound system (Philips Healthcare, Bothell, WA, USA) was used to assess the presence of plaques in 2 vascular territories (carotid and femoral) at both sides, right and left, which were considered together as a single site, as both side share similar hemodynamics. Ultrasound images were acquired with linear high-frequency 2-dimensional probes (Philips Transducer L9-3, Philips Healthcare), using the Bioimage Study protocol for the carotid arteries [17] and a specifically designed protocol for the femoral arteries [18]. A plaque was defined as a focal structure that protrudes into the lumen of the carotid artery at least 0.5 mm or ≥50% thicker than the surrounding intima-media thickness. All measurements were analyzed using electrocardiogram gated frames corresponding to end-diastole (R-wave) [19]. Presence of subclinical atherosclerosis was defined as the presence of at least 1 plaque in any of the 2 vascular territories.

### 2.3. Physical Activity and Sedentary Assessment

To estimate sedentary time, we used the question “How many hours do you usually spend sitting or reclining in a typical working day?”, with values ranging from “never” to “nine or more than nine hours a day,” and that took into account both working and leisure time in a typical working day. In the analysis, the sample was divided into two groups: those who reported being seated <9 h/day, and those who reported being seated ≥9 h/day. Physical activity was assessed using the validated Spanish version [20] of the questionnaire on the frequency of engaging in physical activity used in the Nurses’ Health Study [21] and the Health Professionals’ Follow-up Study [22]. To compute the volume of activity performed by each participant, a metabolic cost was assigned to each activity using Ainsworth’s compendium for physical activities [23], and multiplied by the time the participant reported practicing that activity. From the sum of all activities, we obtained a value of overall weekly METs-h.

### 2.4. Demographic, Clinical, and Biochemical Characteristics

Demographic, anthropometric, and clinical data were obtained by trained personnel during the annual medical examination of the manufacturing company. They included age; body mass index (BMI), which was calculated as weight (in kilograms) divided by height (in meters) squared (kg/m^2^); waist circumference; and blood pressure. Likewise, biochemical measurements of total cholesterol, high-density lipoprotein cholesterol (HDL-c), triglycerides, and fasting serum glucose concentrations were determined by enzyme analysis using the ILAB 650 analyzer from Instrumentation Laboratory (Bedford, MA, USA). Blood samples were collected in fasting (>8 h) conditions. Low-density lipoprotein cholesterol (LDL-c) was calculated using the Friedewald formula [24] when the triglyceride levels were <400 mg/dL. In all participants, non-HDL-c was calculated by subtracting the HDL-c value from the total cholesterol. We defined arterial hypertension as having systolic blood pressure ≥140 mmHg, diastolic blood pressure ≥90 mmHg, or self-reported use of antihypertensive medication [25]. Dyslipidemia was defined as having total cholesterol ≥240 mg/dL, LDL-c ≥160 mg/dL, HDL-c <40 mg/dL, or self-reported use of lipid-lowering drugs [26]. Diabetes was defined as fasting plasma ≥126 mg/dL or self-reported treatment with hypoglycemic medication [25]. Smoking habits were categorized as ever smoker (current and former smoker) if the participant reported having smoked in the last year, or having smoked at least 50 cigarettes in his lifetime, and never smoker.

### 2.5. Statistical Analysis

Descriptive statistics were reported as mean, standard deviation, and percentage. Presence of atherosclerotic plaques in carotid arteries, in femoral arteries, or in any of both territories were fitted separately with logistic regression models depending on sitting time and adjusted for age, BMI, hypertension, dyslipidemia, diabetes, smoking status, and physical activity (METs-h/week). Coefficients were used to calculate odds ratios (OR) for plaque presence of each sitting exposure. In particular, ORs for the presence of plaque for each group of sitting time were calculated using as reference the biggest sample group (≥9 h-sitting/day). A model exploring hourly groups suggested that most differences appeared between the last group and the rest, which focused the analyses on this threshold. *P*-values below 0.05 were considered statistically significant. R statistical software (ver. 3.4.4) was used for the analyses (R Foundation for Statistical Computing, Vienna, Austria).

## 3. Results

Among the 2082 AWHS participants (mean age 50.9; SD 3.9 years), 528 declared spending ≥9 h-sitting/day. They had higher BMI and waist circumference, as well as slightly lower LDL-c than those participants who spent <9 h-sitting/day (Table 1).

**Figure 1:**
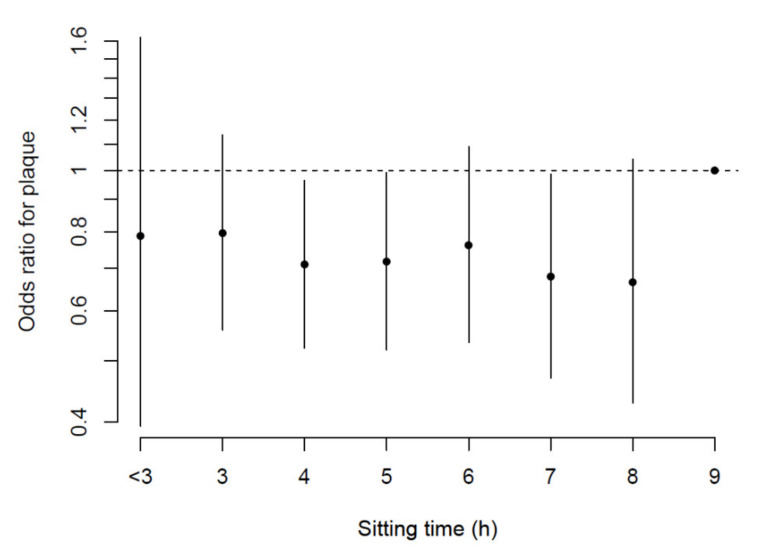
Odds Ratio (95%CI) for the presence of plaque in any of the analyzed territories (carotid and femoral) in terms of total hours of sitting time in the day.

When studying ORs for plaque development in any of the analyzed territories in each group in a disaggregated variable of total hours sitting time per day, at the detail provided by the collecting instrument (hourly detail), a steep difference was apparent at the 9 h threshold (Figure 1). Although a dose-response curve was not apparent, subsequent analyses shown below confirmed this difference.

At least one carotid plaque was present in 764 participants (36.7% of the overall sample), 35.4% in the <9 h-sitting/day group and 40.5% of the ≥9 h-sitting/day group. The odds for having a carotid plaque were 1.25 (95%CI: 1.01, 1.55, *p* < 0.05) times higher among those who spent ≥9 h-sitting/day than among the rest, after adjusting for BMI, diabetes, dyslipidemia, hypertension, smoking habit, and physical activity (Table 2).

Femoral plaques were more frequent as they were present in 1181 participants (56.7% of the overall sample). Although the odds for having a femoral plaque also tended to be higher in the ≥9 h-sitting/day group (Adjusted odds ratio 1.16; 95%CI: 0.93, 1.45, *p* = 0.175), the difference was not statistically significant (Table 2).

Overall, 1400 participants had at least one plaque in any of the territories. A sitting time ≥9 h/day was significantly associated with having at least one plaque (Adjusted odds ratio 1.38; 95%CI: 1.09, 1.74, *p* < 0.01) (Table 2).

## 4. Discussion

In this cross-sectional analysis conducted in young and middle-aged asymptomatic workers, we provide evidence that a lifestyle that implies ≥9 h-sitting/day is associated with higher odds for the presence of subclinical atherosclerosis in the carotid territory, compared with those who were sitting <9 h/day independent of physical activity levels and the presence of other CVD risk factors.

An association between sitting time and CVD risk factors and mortality has been previously described [2,5,7]. However, the association between sitting time and the presence of subclinical atheroma plaques is under-examined, even when it is known that subclinical atherosclerosis constitutes an intermediate process towards clinical CVD and death. In this way, vascular ultrasound is a non-invasively way to assess atherosclerosis and predict major cardiovascular events. Carotid plaque and intima-media thickness (cIMT) measurements are early markers of subclinical atherosclerosis. However, the former is much more relevant, given that a recent research carried out by Sillesen et al. has concluded that cIMT did not improve the risk prediction of major cardiovascular events significantly while atheroma plaque did [27]. Furthermore, in a meta-analysis published by Inaba et al., the ultrasound assessment of carotid plaque has shown to be more accurate than cIMT in the diagnostic of coronary artery disease [19].

Only two studies have evaluated the presence of carotid artery plaque, and they have been carried out in mostly female populations [13,14]. In a recent European study conducted by Lazaros et al., an upward trend was observed between sitting time and the prevalence of carotid atheroma plaque, the mean cIMT, and the maximum cIMT, although no statistic significance was found [13]. Besides, another study conducted in a small sample (*n* = 340) of a Mexican American population [14] did not find any significant association between total sitting time and the presence of carotid plaque, mean cIMT, or cIMT ≥75%. However, when stratified by levels of physical activity, they found association in one of the strata. Thus, with our results, we finally confirm this previously elusive fact that an association of long sitting time with atherosclerosis exists as early as in the stage of subclinical atherosclerosis.

The independency between the levels of physical activity and sitting time has been debated [28,29]. Several prospective studies suggest that physical activity may not necessarily undo the harms from excessive sitting time [2], but more recent studies on mortality have shown a reduced influence of sitting time at higher amounts of physical activity, and vice-versa, a reduced influence of physical activity among those that spend less time sitting [5,28,29]. All these studies, which performed dose-response analysis, coincided that for above 8 h/day of sitting time there was a substantial increase in the risk, except for those at higher levels of physical activity where this risk did not increase. However, that amount of physical activity is likely to be performed by only a small proportion of the general population, and it is likely to be worthwhile to act independently of physical activity. In the present study, the average level of physical activity performed by our sample was 32.8 METs-h/week [30], below the levels where it has been shown to counteract the influence of sitting time on mortality. Thus, we could indicate that with more detailed methods, we are able to show that there is a relationship between sitting time and the presence of subclinical atherosclerosis, which seems independent from physical activity in the range performed by the general population. Using this intermediate endpoint may have been essential to the demonstration because demonstrating an effect on mortality requires extremely big samples and long follow-ups and it is obscured by the sum of several other influences.

It is remarkable that we found a more intense association in the carotid territory than in the femoral one, while at the age of our sample participants the femoral territory is more affected by atherosclerosis and its presence is more intensely associated with traditional risk factors [31]. The extent to which atherosclerosis affects different sites depends on several factors such as the genetic background, immune status, gender, and oxidative stress, among others [32]. Our results hinted that longer sitting time was associated more with carotid atherosclerosis than with femoral atherosclerosis. Atherosclerosis susceptibility is conditioned by hemodynamic features, which play a major role in the localization of atherosclerosis lesions [32]. Several studies showed that body position affects hemodynamics [8,33,34], and erect posture might play a role in the atherogenesis of leg arteries [33,35], so the sitting position could modify hemodynamics, assumedly protecting leg arteries, and therefore, justify our findings. Another possible explanation is that the greater association of traditional risk factors on femoral arteries atherosclerosis blurs the effect of sitting time on that territory.

As opposed to physical activity, which is agreed to be measured in terms of energy expenditure, researchers have used multiple definitions and methods to assess the sedentary behavior. It is usually measured by accelerometry or questionnaires, and it has been defined in the literature in several ways, for example, the duration of all activities that required an energy expenditure at the level of 1.0–1.5 METs during waking hours [36], the total screen time, the total TV-viewing time, or the total sitting time during working and leisure time in a day or a week [37]. This last definition, and no other sedentary behavior definitions that involve non-fatiguing muscle contractions such as standing position, is associated with a reduced contractile stimulation activity in postural skeletal muscles, causing a decrease of lipoprotein lipase enzyme activity [3,4,38], limiting the uptake of triglycerides and free fatty acid, and also reducing HDL-cholesterol plasma concentration [38] and promoting a proinflammatory state [10].

This study has the strength of a reasonable sample size and the use of high-quality data collection methods to obtain information on subclinical atherosclerosis and other variables. Besides, we believe that missing data did not create selection bias because there were no statistically significant differences in the sitting time distribution between participants excluded and those analyzed. However, several limitations should be acknowledged in our study. First, the cross-sectional analysis does not allow us to establish a causal temporal link between sitting time and the presence of subclinical atheroma plaque, although being subclinical it is unlikely to be responsible of reverse causation. Second, the sample includes only men, and therefore, our results may not be generalizable to women. Third, although personal interviews to collect data about sitting time and physical activity have been carried out by trained interviewers, the use of self-reported information could be subject to bias.

In conclusion, sitting ≥9 h/day is associated with higher odds for carotid and any territory plaque development independently of physical activity levels and other CVD risk factors.

## Figures and Tables

**Table 1:** Baseline characteristics of study participants according to sitting time category.

Variable	Overall	<9 h/day	≥9 h/day	*p*-Value
*n*	2082	1554	528	
Age, years	50.9 (3.9)	50.8 (3.9)	51.0 (3.9)	0.353
BMI, kg/m^2^	27.6 (3.3)	27.5 (3.2)	28.0 (3.4)	0.007
Waist circumference, cm	97.3 (8.9)	96.9 (8.8)	98.4 (8.8)	0.001
Systolic blood pressure, mmHg	125.4 (13.9)	125.4 (14.0)	125.4 (13.7)	0.941
Diastolic blood pressure, mmHg	82.4 (9.4)	82.4 (9.5)	82.4 (9.2)	0.899
Total cholesterol, mg/dL	220.1 (36.4)	220.9 (36.5)	217.7 (36.1)	0.084
HDL-c, mg/dL	53.0 (11.4)	53.2 (11.3)	52.6 (11.5)	0.299
Non-HDL-c, mg/dL	167.1 (35.2)	167.7 (35.2)	165.1 (35.3)	0.146
LDL-c, mg/dL	137.9 (31.4)	138.8 (31.2)	135.4 (31.8)	0.034
Triglycerides, mg/dL	150.1 (97.1)	148.8 (97.1)	153.9 (97.1)	0.303
Glucose, mg/dL	97.7 (17.5)	97.4 (16.5)	98.8 (20.1)	0.092
Hypertension, %	37.5 (781)	37.3 (580)	38.1 (201)	0.760
Dyslipidemia, %	49.2 (1025)	48.8 (758)	50.6 (267)	0.477
Diabetes, %	5.6 (117)	5.1 (79)	7.2 (38)	0.076
Ever smokers, %	77.1 (1606)	76.9 (1195)	77.8 (411)	0.655

BMI: body mass index; HDL-c: High-density lipoprotein cholesterol. Values are mean (SD) or % (number).

**Table 2:** Odds ratio (95%CI) for the presence of plaque in different territories by sitting time category.

Different Models in the Analyzed Territories	Sitting Time		*p*-Value
	<9 h/day	≥9 h/day	
Number with carotid plaque/Total	550/1554	214/528	
Age-adjusted	1.00 (ref)	1.23 (0.99, 1.51)	0.051
Multivariable-adjusted 1	1.00 (ref)	1.24 (1.00, 1.53)	0.047
Multivariable-adjusted 2	1.00 (ref)	1.25 (1.01, 1.55)	0.037
Number with femoral plaque/Total	868/1554	313/528	
Age-adjusted	1.00 (ref)	1.13 (0.92, 1.39)	0.231
Multivariable-adjusted 1	1.00 (ref)	1.14 (0.92, 1.42)	0.237
Multivariable-adjusted 2	1.00 (ref)	1.16 (0.93, 1.45)	0.175
Number with plaque in any territory/Total	1020/1554	380/528	
Age-adjusted	1.00 (ref)	1.33 (1.07, 1.67)	0.010
Multivariable-adjusted 1	1.00 (ref)	1.35 (1.07, 1.71)	0.010
Multivariable-adjusted 2	1.00 (ref)	1.38 (1.09, 1.74)	0.007

Model 1 adjusted for age, body mass index, hypertension, dyslipidemia, diabetes, and smoking status. Model 2 additionally adjusted for physical activity (METs-h/week); (ref): Reference.

## Data Availability

The data presented in this study are available on request from the corresponding author. The data are not public due to ethical reasons.
